# CRISPRi-Mediated Epigenetic Suppression of TERT Reduces Cell Growth in Non-Small-Cell Lung Cancer Cells

**DOI:** 10.3390/cells15131150

**Published:** 2026-06-24

**Authors:** Seong-Ho Park, Juyoung Hong, Woochang Hwang, Minjeong Kim, Hyeon Jong Yu, Taegeun Bae, Hyomin K. Lee, Ji Yeoun Lee, Young Chan Lee, Chul-Kee Park, Junho K. Hur

**Affiliations:** 1 Department of Life Science, College of Natural Sciences, Hanyang University, Seongdong-gu, Seoul 04763, Republic of Korea; spark94@hanyang.ac.kr; 2 Department of Biomedical Science, Graduate School of Biomedical Science and Engineering, Hanyang University, Seoul 04763, Republic of Korea; juyoung@hanyang.ac.kr (J.H.); juhur@hanyang.ac.kr (J.K.H.); 3 Hanyang Institute of Bioscience and Biotechnology, Hanyang University, Seoul 04763, Republic of Korea; 4 Department of Pre-Medicine, College of Medicine, Hanyang University, Seoul 04763, Republic of Korea; 5 Department of Medicine (AgeTech-Service Convergence Major), College of Medicine, Kyung Hee University, Seoul 02447, Republic of Korea; sicm@khu.ac.kr; 6 Department of Neurosurgery, Seoul National University College of Medicine, Seoul National University Hospital, 101 Daehak-ro, Jongno-gu, Seoul 03080, Republic of Korea; hjyu1357@snu.ac.kr (H.J.Y.); nsckpark@snu.ac.kr (C.-K.P.); 7 Seoul National University College of Medicine, Seoul 03080, Republic of Korea; 8 Hanyang Biomedical Research Institute, Hanyang University, Seoul 04763, Republic of Korea; btg417@naver.com (T.B.); lumiere55@hanyang.ac.kr (H.K.L.); 9 Department of Genetics, College of Medicine, Hanyang University, Seoul 04763, Republic of Korea; 10 Department of Anatomy and Cell Biology, Seoul National University College of Medicine, Seoul 03080, Republic of Korea; ddang1@snu.ac.kr; 11 Division of Pediatric Neurosurgery, Seoul National University Children’s Hospital, Seoul 03080, Republic of Korea; 12 Neuroscience Research Institute, Medical Research Center, Seoul National University College of Medicine, Seoul 03080, Republic of Korea; 13 Department of Otolaryngology-Head and Neck Surgery, Kyung Hee University School of Medicine, Kyung Hee University Hospital at Gangdong, Seoul 05278, Republic of Korea

**Keywords:** TERT, telomerase, CRISPR interference, dCas9-KRAB, transcriptional repression, cancer cells, gene regulation

## Abstract

**Highlights:**

**What are the main findings?**

- CRISPR inhibition by fusion of catalytically inactive dCas9 and transcription repressor KRAB (dCas9-KRAB) effectively reduced *TERT* expression with growth-inhibitory effects observed in H1299 non-small-cell lung cancer cell line.
- Unlike conventional CRISPR methods that induce changes in DNA sequences, dCas9-KRAB epigenetically repressed the expression of *TERT* at transcription level, without genome editing at DNA level.

**What are the implications of the main findings?**

- Compared with RNA-targeting CasRx, dCas9-KRAB-mediated targeting elicited weaker innate immune and stress responses under the tested conditions in H1299 cells.
- dCas9-KRAB-mediated CRISPRi may provide a proof-of-principle framework for gene-regulation-based approaches to TERT-dependent cancers.

**Abstract:**

TERT, the catalytic subunit of telomerase, is aberrantly activated in most cancers and represents an attractive therapeutic target. However, conventional *TERT*-targeting strategies, including chemical inhibitors and siRNA, are limited by several issues, such as insufficient efficacy and off-target effects. In this study, we investigated whether dCas9-KRAB-mediated CRISPR interference (CRISPRi) could overcome the limitations by transcriptional repression of *TERT* without DNA cleavage. We first assessed the efficacy of the dCas9-KRAB system by applying it to H1299 non-small-cell lung cancer cells and observed reduction in *TERT* expression up to approximately 80% and significant decreases in cell viability and growth. Transcriptome-wide analysis showed limited detectable changes in non-target-gene expression under the conditions tested. Together, the results suggest that dCas9-KRAB-mediated CRISPRi could serve as a proof-of-principle approach for targeted repression of *TERT* in cancer cells with limited detectable effects on non-target-gene expression.

## 1. Introduction

Telomeres are repetitive DNA sequences located at chromosome ends that preserve genomic stability. In normal somatic cells, telomeres shorten progressively with each cell division, ultimately leading to replicative senescence or apoptosis [1]. In contrast, many cancer cells maintain telomere length through activation of telomere maintenance mechanisms, thereby overcoming these proliferative barriers [2]. Telomerase plays a central role in this process, and its catalytic subunit, telomerase reverse transcriptase (*TERT*), is reactivated in approximately 90% of human cancers [1,3]. *TERT* activation is frequently driven by promoter mutations and epigenetic alterations, including aberrant promoter methylation [4,5,6]. Beyond promoter mutations, *TERT* expression is also modulated through promoter-proximal regulatory variants and transcription factor binding; for example, a *TERT* promoter variant has been shown to regulate *TERT* expression via androgen signaling-orchestrated binding of MYC and E2F1 in prostate cancer [7]. These observations underscore the importance of promoter-proximal and regulatory mechanisms in *TERT* activation, supporting the rationale for targeting this region with CRISPRi. TERT is a central regulator of telomere maintenance and contributes to the replicative immortality of cancer cells, making it an attractive therapeutic target [8]. In contrast, a subset of cancers maintains telomeres through the alternative lengthening of telomeres (ALT) pathway, which functions independently of TERT. U2OS cells are a well-established ALT-positive cell line and therefore provide a useful negative control for evaluating TERT-dependent phenotypes.

A variety of therapeutic strategies have been developed to inhibit telomerase or TERT. Chemical inhibitors such as BIBR1532 have shown potential to suppress cancer cell growth through telomerase inhibition [9,10], but their utility may be limited by insufficient efficacy under non-toxic conditions [11]. siRNA-based approaches have been used to reduce *TERT* expression [12,13], yet their utility is limited by off-target effects [14,15]. In addition, because these approaches often fail to sustain *TERT* suppression, repeated administration may be necessary to maintain their effects.

Although conventional CRISPR-Cas9 enables efficient gene disruption, its reliance on DNA cleavage and permanent genomic modification raises concerns about unintended off-target alterations, immunogenic responses, and therapeutic safety [16,17,18]. In parallel, nuclease-deficient CRISPR platforms have been developed to modulate endogenous gene expression without introducing DNA double-strand breaks [19]. Among these approaches, CRISPR interference (CRISPRi) offers an attractive strategy for repressing gene expression at the transcriptional level. CRISPRi uses catalytically inactive Cas9 (dCas9) fused to a transcriptional repressor to silence target genes at the transcriptional level [20,21]. This approach is particularly effective when directed to promoter or transcription start site proximal regions [21,22]. Among CRISPRi platforms, dCas9-KRAB has been widely used because the KRAB domain recruits transcriptional repressors and induces robust gene silencing [20,23]. These features make dCas9-KRAB-mediated CRISPRi a potentially useful platform for sustained repression of cancer-associated genes.

However, whether dCas9-KRAB-mediated CRISPRi can achieve sustained repression of *TERT* with limited non-target cellular responses remains insufficiently characterized. In this study, we investigated whether dCas9-KRAB-mediated CRISPRi could effectively suppress *TERT* expression and inhibit the growth of TERT-expressing cancer cells. Beyond demonstrating repression efficacy, we also compared the cellular response profiles of dCas9-KRAB and CasRx (RfxCas13d), an RNA-targeting CRISPR-Cas13d system, with a focus on innate immune activation and cellular stress responses, factors that are particularly relevant for applications requiring sustained gene repression. We further included a transcriptome-wide analysis to explore whether apparent non-target transcriptional changes accompanied dCas9-KRAB-mediated *TERT* repression.

## 2. Materials and Methods

### 2.1. Plasmid Construction

A lentiviral vector expressing dCas9-KRAB-mediated CRISPR interference (CRISPRi) was constructed using lentiCRISPR v2 (Addgene plasmid #52961) and a dCas9-KRAB donor plasmid (Addgene plasmid #110820). To generate the lentiviral backbone, the EF1α promoter-containing lentiCRISPR v2 vector was amplified by PCR while excluding the Cas9 coding region. The dCas9-KRAB fragment was amplified separately and assembled into the lentiviral backbone using NEBuilder HiFi DNA Assembly Master Mix (New England Biolabs, Ipswich, MA, USA) according to the manufacturer’s instructions.

For sgRNA cloning, the vector was digested with BsmBI (New England Biolabs, Ipswich, MA, USA). Because a BsmBI recognition site was present within the dCas9-KRAB sequence, a silent mutation was introduced to eliminate this site without altering the encoded amino acid sequence. The final construct was verified by Sanger sequencing.

### 2.2. Cell Culture and Transfection

HEK293T cells were obtained from ATCC (CRL-3216). HeLa cells were obtained from the Korean Cell Line Bank (KCLB, Seoul, Republic of Korea; KCLB No. 10002). KMH-2 cells were obtained from JCRB Cell Bank (Osaka, Japan; JCRB1066). SNU-4098 (KCLB No. 04098) and SNU-4210 (KCLB No. 04210) were obtained from the Korean Cell Line Bank (KCLB). NCI-H1299 cells were obtained from the Korean Cell Line Bank (KCLB No. 25803), and U2OS cells were obtained from the Korean Cell Line Bank (KCLB No. 30096). All cell lines were obtained from established repositories (ATCC, KCLB, or JCRB) that perform STR-based authentication at the point of distribution. All cell lines were routinely monitored for mycoplasma contamination using the e-Myco Mycoplasma PCR Detection Kit ver.2.0 (iNtRON Biotechnology, Seongnam, Republic of Korea; Cat No. 25235), and tested negative for mycoplasma.

HEK293T and HeLa cells were cultured in DMEM; KMH-2 cells in a 1:1 mixture of DMEM and RPMI-1640; U2OS, SNU-4098, and SNU-4210 cells in RPMI-1640 supplemented with L-glutamine; and NCI-H1299 cells in RPMI-1640. All media were supplemented with 10% fetal bovine serum (FBS; Welgene Inc., Gyeongsan, Republic of Korea) and 1% penicillin/streptomycin (Welgene Inc., Gyeongsan, Republic of Korea). Cells were maintained at 37 °C in a humidified incubator with 5% CO_2_. For transient transfection, cells were seeded at a density of 1 × 10^5^ cells per well in 24-well plates and transfected the following day using Lipofectamine 2000 (Thermo Fisher Scientific, Waltham, MA, USA) according to the manufacturer’s instructions. A total of 1 μg plasmid DNA and 2 μL Lipofectamine 2000 were used per well. Unless otherwise indicated, cells were harvested 48 h after transfection. The sequences of gRNA oligos used in this study are provided in Supplementary Table S1.

### 2.3. Lentivirus Production and Transduction

Lentiviral particles were produced in HEK293T cells using a three-plasmid packaging system consisting of psPAX2 (Addgene plasmid #12260), pMD2.G (Addgene plasmid #12259), and the transfer vector encoding dCas9-KRAB and sgRNA. HEK293T cells were seeded in 10 cm dishes at approximately 60–70% confluence one day before transfection.

For each dish, 10 μg transfer plasmid, 7.5 μg psPAX2, and 2.5 μg pMD2.G were diluted in 3 mL Opti-MEM (Gibco, Thermo Fisher Scientific, Waltham, MA, USA). Lipofectamine 2000 was added at a DNA-to-reagent ratio of 1:2 (*w*/*w*), and the mixture was incubated at room temperature for 15 min before being added to the cells. The culture medium was replaced 24 h after transfection. Viral supernatants were collected at 72 h post-transfection and filtered through a 0.45 μm PES filter.

Filtered viral supernatants were concentrated using Lenti-X Concentrator (Takara Bio/Clontech, CA, USA; catalog no. 631232) according to the manufacturer’s protocol, followed by centrifugation at 4000 rpm for 1 h at 4 °C. Concentrated lentiviral particles were resuspended in Opti-MEM and stored at −80 °C until use.

For lentiviral transduction, target cells were infected with concentrated viral particles at a multiplicity of infection (MOI) of 0.8 under the indicated experimental conditions. The non-targeting (NT) control consisted of cells transduced with the dCas9-KRAB vector without any cloned sgRNA, thereby serving as a no-guide dCas9-KRAB lentiviral control. Following transduction, cells were subjected to puromycin selection to enrich transduced cells (HeLa, 3 μg/mL; H1299 and U2OS, 2 μg/mL; 6 days), followed by a 3-day recovery period before subsequent experiments. Non-transduced cells were eliminated during puromycin selection, and all subsequent analyses were performed using bulk populations rather than individual clones. Cells were harvested at the indicated time points for downstream analyses: RT-qPCR at days 13, 23, and 33; Western blot and transcriptomic analysis samples at day 33; CCK-8 and cell counting at day 18 after transduction.

### 2.4. RNA Extraction and RT-qPCR

Total RNA was extracted using TRIzol reagent (Invitrogen, Carlsbad, CA, USA) according to the manufacturer’s instructions. Briefly, cells were lysed in 500 μL TRIzol per sample, and total RNA was isolated following the standard protocol. RNA concentration and purity were assessed using a NanoDrop spectrophotometer (Thermo Fisher Scientific, Waltham, MA, USA).

For cDNA synthesis, 500 ng total RNA was reverse-transcribed in a 20 μL reaction using the High-Capacity cDNA Reverse Transcription Kit (Applied Biosystems, Foster City, CA, USA). Quantitative PCR was performed using THUNDERBIRD™ Next SYBR^®^ qPCR Mix (TOYOBO, Osaka, Japan) on a CFX Connect Real-Time PCR System (Bio-Rad, Hercules, CA, USA). Each 20 μL reaction contained 1 μL cDNA and 10 pmol each of forward and reverse primers. Primer sequences are listed in Supplementary Table S1.

The thermal cycling conditions were as follows: initial denaturation at 95 °C for 2 min, followed by 40 cycles of 95 °C for 5 s and 60 °C for 30 s. Relative mRNA expression levels were calculated using the 2^−ΔΔCt^ method, with *GAPDH* as the endogenous control, unless otherwise indicated. Expression data are presented as relative expression levels, while statistical analyses were performed using ΔCt values. For the long-term lentiviral RT-qPCR experiments, expression levels were normalized to *GAPDH* and presented relative to the mock group at each time point, which was set to 1. In the corresponding Supplementary Figure S3, the same data are presented as 2^−ΔCt^ values to allow comparison of absolute baseline *TERT* expression levels across cell lines.

### 2.5. Western Blot Analysis

Cells were lysed in RIPA buffer containing 50 mM Tris-HCl (pH 7.4), 150 mM NaCl, 1% NP-40, 0.5% sodium deoxycholate, and 0.1% SDS (Invitrogen, Thermo Fisher Scientific, Carlsbad, CA, USA) supplemented with protease and phosphatase inhibitor cocktails (Invitrogen, Thermo Fisher Scientific, Carlsbad, CA, USA). Lysates were incubated on ice for 30 min and centrifuged at 14,000 rpm for 15 min at 4 °C. The supernatants were collected, and protein concentrations were measured using a BCA Protein Assay Kit (Invitrogen, Thermo Fisher Scientific, Carlsbad, CA, USA).

Equal amounts of protein (10 μg) were separated by 10% SDS-PAGE and transferred onto PVDF membranes (Millipore, Burlington, MA, USA) using a wet transfer system. Membranes were blocked with 5% non-fat milk (BD Difco, Franklin Lakes, NJ, USA) in TBST for 1 h at room temperature and incubated overnight at 4 °C with anti-TERT antibody (Abcam, Cambridge, UK, ab32020; clone Y182; 1:1000; RRID: AB_778263) diluted in 5% milk/TBST. After washing, membranes were incubated with HRP-conjugated goat anti-rabbit IgG antibody (Abcam, Cambridge, UK, ab6721; 1:5000; RRID: AB_955447) for 1 h at room temperature. GAPDH was detected using an anti-GAPDH antibody (rabbit monoclonal, Abcam, Cambridge, UK, ab181602; clone EPR16891; 1:2000; RRID: AB_2630358).

Protein bands were visualized using an enhanced chemiluminescence reagent (Thermo Fisher Scientific, Carlsbad, CA, USA) and imaged using an Amersham Imager 600 (Cytiva, Marlborough, MA, USA). Band intensities were quantified using Fiji (ImageJ, NIH; ImageJ v1.54), and TERT protein levels were normalized to GAPDH. Representative immunoblot data are shown in Supplementary Figure S4.

### 2.6. Cell Viability and Cell Counting Assays

Cell viability was assessed using the Cell Counting Kit-8 (CCK-8) assay (Dojindo Molecular Technologies, Tokyo, Japan) according to the manufacturer’s instructions. Cells were seeded in 96-well plates at a density of 1 × 10^4^ cells per well in 100 μL complete medium and incubated for 24 h to allow attachment. Subsequently, 10 μL of CCK-8 reagent was added to each well, and the plates were incubated for 2 h at 37 °C. Absorbance was measured at 450 nm using a microplate reader (Bio-Rad, Hercules, CA, USA). For cell counting assays, cells were seeded at a density of 2 × 10^4^ cells per well. Cells were counted using an automated cell counter. The cell counting assay was performed with two biological replicates.

### 2.7. RNA Sequencing and Differential Expression Analysis

To assess transcriptomic changes associated with *TERT* repression, RNA-seq was performed using H1299 cells transduced with either a non-targeting sgRNA control or a *TERT*-targeting sgRNA. Two biological replicates were analyzed for each group. mRNA was isolated from 1 μg of total RNA using the NEBNext Poly(A) mRNA Magnetic Isolation Module (New England Biolabs, Ipswich, MA, USA). RNA-seq libraries were prepared using the NEBNext Ultra II Directional RNA Library Prep Kit for Illumina (New England Biolabs, Ipswich, MA, USA) according to the manufacturer’s instructions and indexed using NEBNext Multiplex Oligos for Illumina (Dual Index Primers Set 1) (New England Biolabs, Ipswich, MA, USA). Library concentration was measured using a Qubit fluorometer (Thermo Fisher Scientific, Carlsbad, CA, USA), and library size distribution was evaluated using a TapeStation 2200 (Agilent Technologies, Santa Clara, CA, USA). Sequencing was performed on an Illumina NovaSeq 6000 platform using paired-end reads. RNA samples were collected at day 33 after lentiviral transduction. Differential expression analysis was performed in R (version 4.5.1) using DESeq2 (v1.48.2) after prefiltering genes with counts ≥ 10 in at least two samples to remove features with limited information for stable inference. Shrunken log2 fold changes were estimated using the lfcShrink function with the apeglm method (apeglm v1.30.0). Volcano plots display −log10(FDR) on the *y*-axis, and statistical significance was defined using Benjamini–Hochberg false discovery rate (FDR) < 0.05 together with an absolute shrunken log2 fold change ≥ 1.

### 2.8. Statistical Analysis

Data are presented as mean ± SEM. Statistical analyses were performed using GraphPad Prism 8 and R (version 4.5.1). For comparisons involving three or more groups, one-way or two-way ANOVA followed by post hoc multiple comparisons testing or unpaired *t*-test was used, as indicated in the corresponding figure legends. Statistical significance was defined as * *p* < 0.05. Exact significance levels are indicated in the corresponding figure legends. For the initial RT-qPCR screening and validation experiments (transient transfection), data were obtained from three biological replicates. For the long-term lentiviral RT-qPCR experiments, data were obtained from two biological replicates. Transcriptomic analysis was based on two biological replicates. The cell counting assay at day 18 was performed with two biological replicates, and the assay at day 65 was performed with three biological replicates. Technical triplicates were used for RT-qPCR, whereas no technical replicates were included for the cell counting assays. Details for each experiment are provided in the corresponding figure legends.

## 3. Results

### 3.1. dCas9-KRAB Efficiently Represses TERT Across Several Cancer Cell Lines

**Figure 1:**
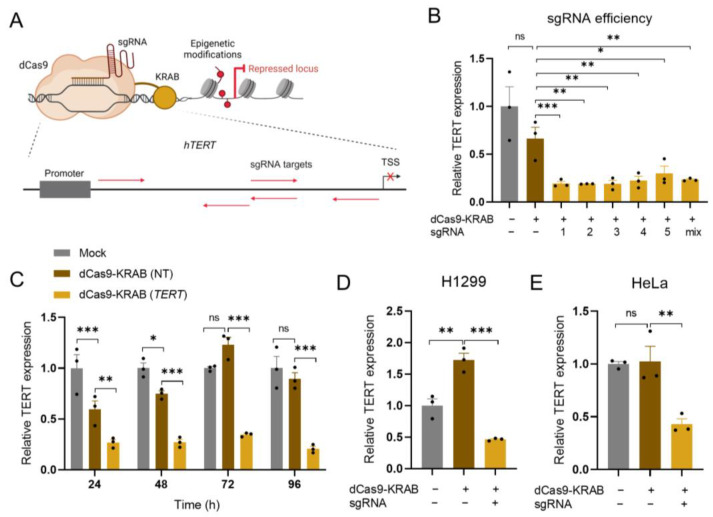
Screening and validation of dCas9-KRAB-mediated *TERT* repression across several cell lines. (**A**) Schematic illustration of sgRNA design targeting regions proximal to the transcription start site (TSS) within the *TERT* promoter. Arrows indicate the individual sgRNAs, and the dashed lines indicate the magnified region around the TSS of the *hTERT* promoter. The schematic is not to scale. (**B**) Repression efficiency of individual sgRNAs and a mixture of all five sgRNAs was evaluated by RT-qPCR in HEK293T cells after targeting *TERT* with dCas9-KRAB. (**C**) Time course analysis of *TERT* mRNA repression in HEK293T cells from 24 to 96 h after transfection. (**D**,**E**) RT-qPCR analysis of *TERT* mRNA levels in H1299 (**D**) and HeLa (**E**) following dCas9-KRAB-mediated repression. *TERT* mRNA levels were normalized to *GAPDH* and presented relative to the mock control, which was set to 1. Data are presented as mean ± SEM from three biological replicates. Statistical significance was assessed by one-way ANOVA followed by a post hoc multiple comparisons test (* *p* < 0.05, ** *p* < 0.01, *** *p* < 0.001; ns, not significant). In panels (**B**–**E**), statistical comparisons were performed for mock vs. dCas9-KRAB (NT) and dCas9-KRAB (NT) vs. dCas9-KRAB (*TERT*).

Previous studies have shown that CRISPR interference (CRISPRi) is most effective when guide RNAs are positioned near the transcription start site (TSS), and that simultaneous targeting of multiple sites can further enhance repression efficiency [24]. Accordingly, five sgRNAs targeting regions proximal to the TSS within the *TERT* promoter were designed using the Cas-Designer web tool (http://www.rgenome.net/cas-designer/, accessed on 22 June 2026) (Figure 1A). To evaluate their activity, the individual sgRNAs as well as a mixture of all five sgRNAs were tested in HEK293T cells after targeting *TERT* with dCas9-KRAB. All five individual and the pooled sgRNAs significantly reduced *TERT* mRNA expression relative to the NT control, with robust and comparable knockdown efficiency (Figure 1B). To assess the temporal stability of repression, *TERT* mRNA levels were monitored from 24 to 96 h after transfection in HEK293T cells, and significant repression was maintained throughout this period (Figure 1C).

We next examined whether dCas9-KRAB-mediated *TERT* repression could be reproduced across cancer cell lines. Robust reduction in *TERT* mRNA levels was observed in H1299 and HeLa cells after dCas9-KRAB targeting (Figure 1D,E). Additional validation confirmed significant *TERT* repression in the anaplastic thyroid carcinoma cell line KMH-2 and in two patient-derived glioblastoma cell lines, SNU-4098 and SNU-4210 (Supplementary Figure S1). Across all tested cell lines, dCas9-KRAB-mediated targeting significantly reduced *TERT* expression. Of note, the effector-only (dCas9-KRAB, no sgRNA) condition altered TERT expression relative to mock in a cell-line-dependent manner, with a modest decrease in HEK293T cells (Figure 1C) and a modest increase in H1299 cells (Figure 1D), whereas no significant change relative to mock was observed in the other cell lines tested (HeLa, Figure 1E; KMH-2, SNU-4098, and SNU-4210, Supplementary Figure S1). As this sgRNA-independent effect was not directionally consistent across cell lines, it likely reflects a general cellular response to transfection rather than a guide-directed effect; in all cases, sgRNA-directed targeting produced robust TERT repression relative to the effector-only condition. Together, these results demonstrate that dCas9-KRAB efficiently represses *TERT* expression across several TERT-expressing cancer cell lines.

### 3.2. dCas9-KRAB-Mediated TERT Repression Is Associated with Attenuated Innate Immune and Stress Responses Relative to CasRx

**Figure 2:**
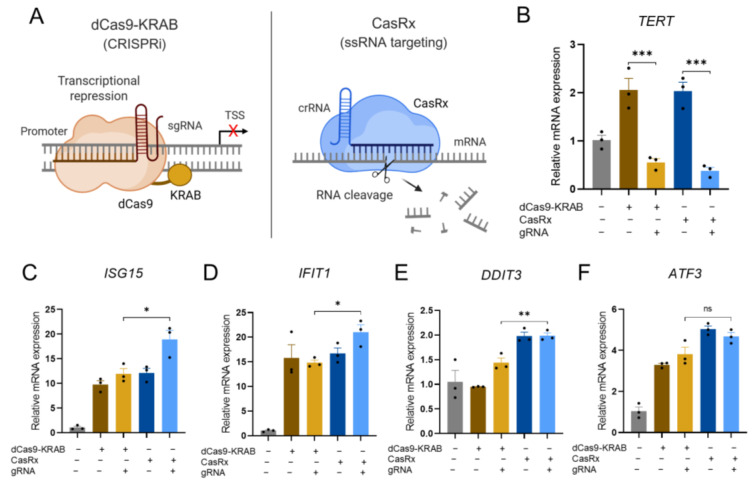
dCas9-KRAB-mediated *TERT* repression is associated with attenuated innate immune and stress responses relative to CasRx. (**A**) Schematic illustration of the mechanisms of action of dCas9-KRAB and CasRx. dCas9-KRAB represses gene expression through KRAB-mediated epigenetic silencing at the DNA level, whereas CasRx mediates target RNA cleavage. (**B**) RT-qPCR analysis of *TERT* expression in H1299 cells following dCas9-KRAB- or CasRx-mediated targeting. (**C**–**F**) RT-qPCR analysis of the RNA samples used in panel B showing the expression changes of *ISG15* (**C**), *IFIT1* (**D**), *DDIT3* (**E**), and *ATF3* (**F**). Expression levels were normalized to *GAPDH* and presented relative to the mock, which was set to 1. Data are presented as mean ± SEM from three biological replicates. Statistical significance was assessed by unpaired *t*-test (* *p* < 0.05, ** *p* < 0.01, *** *p* < 0.001; ns, not significant).

Efficient target repression alone does not fully reflect the broader cellular consequences of a gene-silencing system. We therefore sought to assess whether dCas9-KRAB might serve as a more suitable platform for *TERT*-targeting than an alternative CRISPR-based tool, CasRx, by comparing their cellular response profiles under the same experimental conditions. To this end, we compared dCas9-KRAB and CasRx under transient *TERT*-targeting conditions (Figure 2A). For this comparison, sgRNA-1 was selected for dCas9-KRAB based on its repression efficiency in the initial screening. Two CasRx guide RNAs were designed using the Cas13design web tool (https://cas13design.nygenome.org/, accessed on 22 June 2026). Both were tested for *TERT* repression in H1299 cells (Supplementary Figure S2), and guide RNA 1, which produced more efficient repression, was selected for subsequent analysis. In H1299 cells, dCas9-KRAB-mediated targeting and CasRx-mediated targeting both reduced *TERT* expression, indicating that both systems could suppress *TERT* under these conditions (Figure 2B).

To determine whether the two platforms differ in innate immune and cellular stress responses under conditions that produced comparable *TERT* repression, we measured *ISG15, IFIT1, DDIT3*, and *ATF3* expression in H1299 cells using the same RNA samples analyzed for *TERT* expression (Figure 2C–F). *ISG15, IFIT1*, and *DDIT3* were upregulated to a significantly greater extent in the CasRx *TERT*-targeting group than in the dCas9-KRAB *TERT*-targeting group. For *ATF3*, no significant difference was observed between dCas9-KRAB-treated and CasRx-treated cells. Notably, across all four markers, CasRx-treated cells showed a consistent trend toward higher expression than dCas9-KRAB-treated cells. We note, however, that the CasRx-only (guide-free) condition showed a trend toward higher baseline expression of *DDIT3* and *ATF3* than the dCas9-KRAB condition, which should be considered when interpreting this comparison. Taking this into account, these findings indicate that under the tested conditions in H1299 cells, dCas9-KRAB-mediated CRISPRi elicits weaker innate immune and stress responses than CasRx. In this context, dCas9-KRAB-mediated CRISPRi may serve as a useful framework for sustained transcriptional repression of cancer-associated genes.

### 3.3. Lentiviral Delivery Enables Sustained TERT Repression

To examine the long-term effects of *TERT* repression, lentiviral delivery was used to establish sustained dCas9-KRAB-mediated CRISPRi. For these experiments, we generated a one-vector lentiviral system co-expressing dCas9-KRAB and sgRNA-1. sgRNA-1 was selected for subsequent experiments based on its robust repression efficiency in the initial screening.

**Figure 3:**
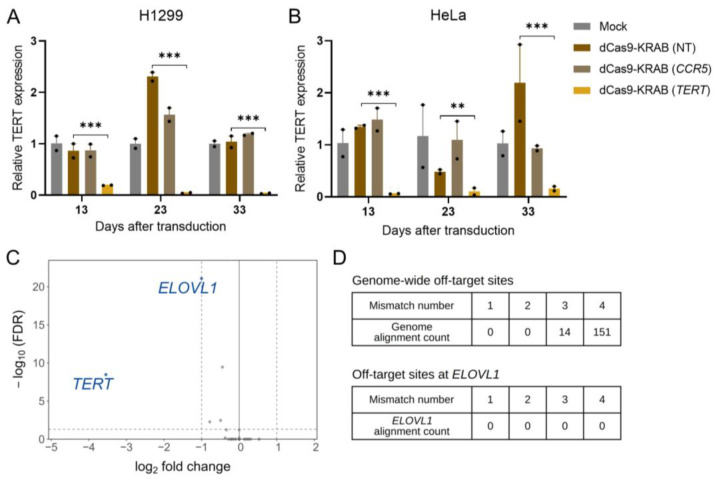
Sustained *TERT* repression by lentiviral dCas9-KRAB with transcriptomic and in silico off-target analyses. (**A**,**B**) RT-qPCR analysis of long-term *TERT* mRNA expression in H1299 (**A**) and HeLa (**B**) cells following lentiviral delivery. Expression levels were normalized to *GAPDH* and are presented relative to the mock group at each time point, which was set to 1. dCas9-KRAB(NT), dCas9-KRAB with non-targeting sgRNA; dCas9-KRAB(*CCR5*), dCas9-KRAB with sgRNA targeting *CCR5*; dCas9-KRAB(*TERT*), dCas9-KRAB with sgRNA targeting *TERT*. Data are presented as mean ± SEM from two biological replicates. Statistical significance was assessed by two-way ANOVA with Dunnett’s post hoc multiple comparisons test. Statistical comparisons were performed between the NT control and the *TERT*-targeting group at each time point. Exact adjusted *p*-values for NT-versus-*TERT*-targeting comparisons: H1299—all time points, *p* < 0.0001. HeLa—day 13, *p* < 0.0001; day 23, *p* = 0.0003; day 33, *p* < 0.0001 (** *p* < 0.01, *** *p* < 0.001). (**C**) Volcano plot of differential gene expression between *TERT*-targeting H1299 cells and non-targeting sgRNA lentiviral control (NT) cells at day 33 after lentiviral transduction. Shrunken log2 fold changes estimated by lfcShrink are shown on the *x*-axis, and −log10(FDR) are shown on the *y*-axis. Genes with FDR < 0.05 and |log2 fold change| ≥ 1 were considered significant; blue dots represent significantly downregulated genes, and gray dots represent non-significant genes (NS). (**D**) In silico off-target prediction using Cas-OFFinder. The top panel displays predicted genome-wide candidate off-target sites, whereas no predicted off-target sites were identified at the *ELOVL1* locus (bottom panel).

*TERT* mRNA levels were monitored in H1299, HeLa, and U2OS cells at days 13, 23, and 33 after lentiviral transduction. In H1299 and HeLa cells, sustained *TERT* repression was observed at all examined time points, with significant reductions in *TERT* expression (Figure 3A,B; Supplementary Figure S3A,B). By contrast, no reduction in *TERT* expression was observed in U2OS cells, likely reflecting the minimal baseline *TERT* expression in these cells (Supplementary Figure S3C). Consistent with the mRNA data, representative immunoblot analysis showed a modest reduction in the apparent TERT band in H1299 cells at day 18 and day 33 after lentiviral transduction, although this protein-level analysis is interpreted with caution given the limitations of the antibody used (Supplementary Figure S4).

To further assess broader molecular consequences of sustained *TERT* repression, RNA-seq was performed in H1299 cells at day 33 after lentiviral transduction. Differential expression analysis showed that *TERT* was the most prominently downregulated transcript. Under the applied analysis thresholds, *ELOVL1* was the only additional downregulated gene identified in the volcano plot (Figure 3C), whereas most genes remained close to the baseline distribution, indicating limited detectable transcriptomic perturbation under these conditions (the full list of differentially expressed genes is provided as Supplementary Data S1).

To provide additional support, RNA-seq quality-control analyses (Supplementary Figure S5) and a mock-versus-*TERT*-targeting comparison were performed (Supplementary Figure S6). In the principal component analysis, the *TERT*-targeting samples clustered relatively closely, whereas the non-targeting control samples showed greater dispersion (Supplementary Figure S5A); given the *n* = 2 design, this is interpreted as a supportive sample-level visualization rather than definitive evidence of treatment-driven clustering. The MA plot showed that the majority of expressed genes were distributed around zero fold change (Supplementary Figure S5B), and the DESeq2 dispersion estimates were consistent with appropriate model fitting (Supplementary Figure S5C). The mock-versus-*TERT*-targeting comparison (Supplementary Figure S6) was consistent with the NT-versus-*TERT*-targeting comparison, and across both, only *TERT* and *ELOVL1* met the significance criteria, with the overall transcriptomic perturbation remaining limited (the full list of differentially expressed genes is provided as Supplementary Data S1). Consistent with this, the innate immune and cellular stress markers examined in the CasRx comparison (*ISG15, IFIT1, DDIT3*, and *ATF3*) showed no significant changes in the lentiviral *TERT*-targeting RNA-seq dataset (Supplementary Table S2), further supporting that sustained dCas9-KRAB-mediated *TERT* repression does not provoke detectable innate immune or stress responses under these conditions.

To examine whether the *ELOVL1* reduction might reflect sgRNA-dependent off-target effects, in silico off-target prediction was performed using Cas-OFFinder (http://www.rgenome.net/cas-offinder/, accessed on 22 June 2026). No candidate off-target sites were identified with one or two mismatches, whereas candidate sites were predicted only at higher mismatch levels. None of these predicted sites mapped to the *ELOVL1* locus (Figure 3D). These findings suggest that the observed reduction in *ELOVL1* is unlikely to be explained by direct sgRNA-dependent off-target binding predicted by sequence similarity alone. Overall, these data indicate that sustained *TERT* repression was accompanied by limited detectable transcriptional changes under the conditions tested. Furthermore, targeting an unrelated genomic locus (*CCR5*) produced no detectable transcriptomic changes relative to mock (Supplementary Figure S7), supporting that the observed effects were not attributable to the lentiviral dCas9-KRAB delivery system itself.

### 3.4. Sustained TERT Repression Impairs Cancer Cell Growth

**Figure 4:**
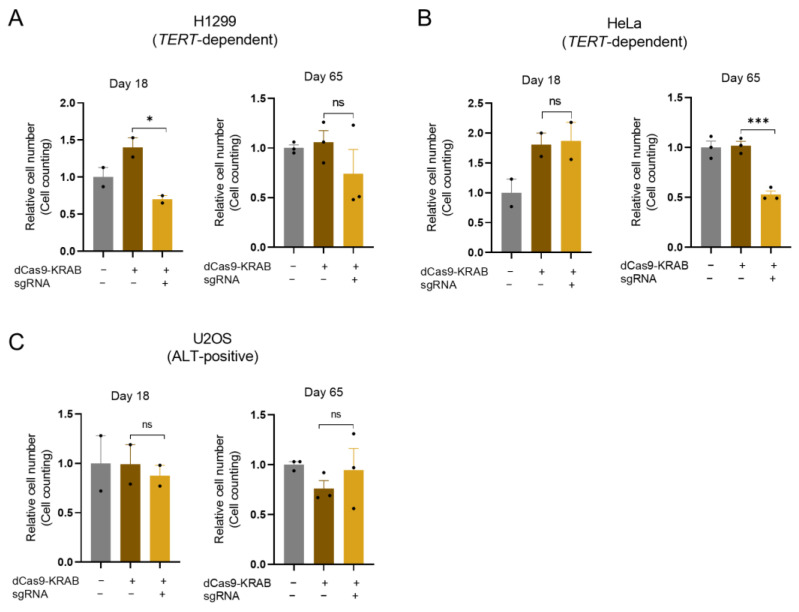
Sustained *TERT* repression impairs cancer cell growth. (**A**–**C**) Cell growth following *TERT* repression was assessed by direct cell counting in H1299 (**A**), HeLa (**B**), and U2OS (**C**) cells at day 18 and day 65 after lentiviral transduction. H1299 and HeLa are TERT-dependent cancer cell lines, whereas U2OS is an ALT-positive cell line that maintains telomeres independently of TERT. Data are presented as mean ± SEM from two biological replicates (day 18) or three biological replicates (day 65). Statistical significance was assessed by one-way ANOVA followed by Dunnett’s post hoc multiple comparisons test (* *p* < 0.05, *** *p* < 0.001; ns, not significant). Exact adjusted *p*-values for NT-versus-*TERT*-targeting comparisons: H1299 day 18, *p* = 0.0459; H1299 day 65, *p* = 0.3809; HeLa day 18, *p* = 0.1520; HeLa day 65, *p* = 0.0006; U2OS day 18, *p* = 0.8977; U2OS day 65, *p* = 0.4634.

To evaluate the functional consequences of prolonged *TERT* repression, cell growth was assessed by direct cell counting at day 18 and day 65 after lentiviral transduction. In H1299 cells, sustained *TERT* repression was associated with significantly reduced cell number at day 18 (Figure 4A), and a trend toward reduced cell number was observed at day 65, although this did not reach statistical significance (*p* = 0.3809). In HeLa cells, a statistically significant reduction in cell number was observed at day 65 (Figure 4B). In contrast, no comparable growth-inhibitory effect was observed in U2OS cells at either time point, consistent with their TERT-independent telomere maintenance mechanism (Figure 4C). CCK-8 assay results for H1299 and U2OS cells are provided in Supplementary Figure S8. These results are consistent with the observed growth-inhibitory phenotype being associated with TERT dependency. Collectively, these findings highlight the feasibility of CRISPRi-based *TERT* repression as a strategy for suppressing the growth of TERT-expressing cancer cells.

## 4. Discussion

In this study, we demonstrated that dCas9-KRAB-mediated CRISPR interference (CRISPRi) effectively represses *TERT* expression in several TERT-expressing cancer cell lines. Using this approach, we achieved substantial repression of *TERT* expression, with the greatest reduction reaching approximately 80%, supporting the feasibility of dCas9-KRAB-mediated *TERT* repression as an approach for functionally targeting *TERT* in cancer cells [25,26]. We also observed that the effector-only (no-sgRNA) dCas9-KRAB condition altered *TERT* expression in a cell-line-dependent manner; this variation may reflect squelching of transcription factors by dCas9-KRAB or a cell-type-specific response to effector expression.

An additional finding of this study is that dCas9-KRAB-mediated *TERT*-targeting was associated with attenuated innate immune and stress responses relative to CasRx under the tested conditions in H1299 cells. Although both systems reduced *TERT* expression, CasRx-mediated targeting induced stronger expression of several response markers, including *ISG15*, *IFIT1*, *DDIT3*, and *ATF3*. This difference may be relevant in settings requiring prolonged gene repression, because excessive activation of stress or immune pathways can complicate the interpretation of downstream phenotypes. In this regard, dCas9-KRAB-mediated CRISPRi may provide a useful framework for sustained transcriptional repression of cancer-associated genes [27]. Validation of this cellular response comparison in additional cell lines will be needed to generalize this observation and is identified as a direction for future studies.

To assess whether dCas9-KRAB-mediated *TERT* repression was associated with broader transcriptional perturbation, RNA-seq analysis was performed in H1299 cells at day 33 after lentiviral transduction. *TERT* was the most prominently downregulated transcript, and *ELOVL1* was the only additional gene meeting the differential expression criteria. Because this analysis was performed with only two biological replicates per group, these transcriptomic findings should be regarded as exploratory and interpreted with caution. Cas-OFFinder analysis did not identify plausible off-target sites at the *ELOVL1* locus, suggesting that direct sgRNA-mediated off-target activity is unlikely, although experimental validation will be required. *ELOVL1* encodes an enzyme involved in the elongation of very long-chain fatty acids, and its potential contribution to the observed growth phenotype warrants investigation in future studies. It is also worth noting that CRISPRi phenotypes can be shaped by local chromatin context and three-dimensional genome organization at the target locus [28], and indirect effects mediated through chromatin reorganization may warrant consideration in future studies. Overall, the available data indicate limited detectable non-target transcriptional changes under the conditions tested.

Beyond the transcriptomic analysis, sustained *TERT* repression was also accompanied by reduced cell growth in TERT-expressing H1299 and HeLa cells, with cell-line-specific kinetics, but not in U2OS cells, which maintain telomeres through alternative lengthening mechanisms. This pattern is consistent with the observed phenotype being associated with TERT dependency. However, because U2OS cells express minimal baseline TERT, they serve as an ALT-positive control comparison rather than as a direct test of *TERT*-repression dependency; the absence of a growth-inhibitory effect in U2OS is therefore interpreted cautiously as evidence against broad non-specific toxicity rather than as direct proof of TERT dependency. The growth effect observed at day 18 further warrants consideration of the underlying mechanism. Because telomere-dependent replicative senescence generally requires progressive telomere shortening over many cell divisions, telomere erosion alone may not fully explain the phenotype observed within this relatively short timeframe. In this context, previous studies have suggested that TERT exerts non-canonical, telomere-independent functions related to cell survival [29], DNA damage responses [30], and mitochondrial protection under oxidative stress [31]. It is therefore possible that suppression of these activities contributed, at least in part, to the early growth-inhibitory effect observed here. However, the mechanism underlying the early growth-inhibitory effect remains unresolved, and direct mechanistic assays will be required to clarify its basis.

In contrast, the growth inhibition observed at the later day 65 time point may be more compatible with telomere-length-dependent mechanisms that require multiple cell divisions to manifest. This was statistically significant in HeLa cells (*p* = 0.0006), whereas H1299 cells showed a non-significant trend toward reduced cell number at day 65 (*p* = 0.3809), consistent with the growth inhibition observed at day 18. However, because telomere length was not directly measured in this study, this possibility remains to be tested.

Several limitations should be considered. First, the present study was performed in cultured cancer cell lines using an integrating lentiviral vector, and as a proof-of-principle study it does not address delivery, reversibility, immunogenicity, potential effects on normal telomerase-positive tissues, or in vivo efficacy; these will require further investigation. Second, while sustained repression of *TERT* expression was confirmed at the mRNA level and supported by representative protein analysis, telomerase activity (e.g., TRAP assay), telomere length dynamics, and additional mechanistic endpoints were not directly assessed. In addition, the anti-TERT antibody used (ab32020, clone Y182) has documented limitations for direct endogenous Western blot detection, and the identity of the detected band in H1299 lysates remains uncertain without further validation; the Western blot data are therefore presented as supportive rather than confirmatory evidence. Furthermore, a TERT rescue experiment using heterologous promoter-driven *TERT* cDNA was not performed, and such an experiment would be required to establish that the observed growth phenotype is specifically attributable to *TERT* repression. Third, the RNA-seq analysis and several growth assays were based on two biological replicates, and these findings should be considered exploratory pending validation in studies with larger sample sizes. Fourth, the sustained lentiviral experiments relied on a single sgRNA (sgRNA-1); validation with a second independent *TERT*-targeting sgRNA in the lentiviral long-term setting would be required to fully establish the causal interpretation and is identified as an important direction for future studies. Fifth, direct quantification of dCas9-KRAB expression across experimental groups was not performed; systematic measurement of effector expression would strengthen the interpretation of repression efficiency comparisons across cell lines. Further studies in more physiologically relevant models will be important to clarify the consequences and potential applications of sustained *TERT* repression.

## 5. Conclusions

This study demonstrates that dCas9-KRAB-mediated CRISPRi enables sustained repression of *TERT* expression in several TERT-expressing cancer cell lines. Compared with CasRx, dCas9-KRAB-mediated targeting showed attenuated innate immune and cellular stress responses, suggesting a more favorable cellular response profile for applications requiring prolonged transcriptional repression. Sustained *TERT* repression was associated with growth-inhibitory effects in H1299 cells, although the mechanistic basis of this phenotype will require further investigation. Taken together, these findings support dCas9-KRAB-mediated *TERT* repression as a proof-of-principle framework for *TERT*-directed gene regulation in cancer.

## Data Availability

The data presented in this study are openly available in BioProject: PRJNA1442345 [http://www.ncbi.nlm.nih.gov/bioproject/PRJNA1442345, accessed on 22 June 2026]. Processed count matrices are available via GEO (accession number: GSE336428). Analysis code is provided as Supplementary Code S1. The full DEG table is provided as Supplementary Data S1.

## Supplementary Materials

The following supporting information can be downloaded at https://www.mdpi.com/article/10.3390/cells15131150/s1. Supplementary Table S1. Sequences of qPCR primers and gRNA oligos used in this study; Supplementary Table S2. Expression of innate immune and cellular stress response genes following sustained *TERT* repression in H1299 cells (RNA-seq); Supplementary Figure S1. RT-qPCR analysis of *TERT* mRNA levels in KMH-2, SNU-4098, and SNU-4210 cells following dCas9-KRAB-mediated repression; Supplementary Figure S2. Comparison of two CasRx guide RNAs for *TERT* repression in H1299 cells; Supplementary Figure S3. Long-term RT-qPCR analysis of *TERT* mRNA expression following lentiviral transduction, presented using two normalization methods; Supplementary Figure S4. Representative immunoblot analysis of TERT protein levels in H1299 cells at day 18 and day 33 after lentiviral transduction; Supplementary Figure S5. RNA-seq quality control of *TERT*-targeting H1299 cells (NT-versus-*TERT*-targeting comparison); Supplementary Figure S6. Additional transcriptomic analysis of *TERT*-targeting H1299 cells (mock-versus-*TERT*-targeting comparison); Supplementary Figure S7. Differential gene expression between *CCR5*-targeting and mock control H1299 cells; Supplementary Figure S8. CCK-8 viability assay following sustained *TERT* repression; Supplementary Code S1. R script for DESeq2 differential expression analysis and visualization; Supplementary Data S1. Full list of differentially expressed genes (DEGs) from the RNA-seq analysis.

## Abbreviations

The following abbreviations are used in this manuscript:

ALT	alternative lengthening of telomeres
ATF3	activating transcription factor 3
CCK-8	Cell Counting Kit-8
CasRx	CRISPR-Cas13d RNA-targeting effector
CRISPR	clustered regularly interspaced short palindromic repeats
CRISPRi	CRISPR interference
dCas9	dead Cas9
DDIT3	DNA damage-inducible transcript 3
DEG	differentially expressed gene
DMEM	Dulbecco’s Modified Eagle Medium
FBS	fetal bovine serum
GAPDH	glyceraldehyde-3-phosphate dehydrogenase
IFIT1	interferon-induced protein with tetratricopeptide repeats 1
ISG15	interferon-stimulated gene 15
KRAB	Krüppel-associated box
NT	non-targeting
PBS	phosphate-buffered saline
PVDF	polyvinylidene difluoride
qPCR	quantitative polymerase chain reaction
RNA-seq	RNA sequencing
RT-qPCR	reverse transcription quantitative polymerase chain reaction
SDS-PAGE	sodium dodecyl sulfate-polyacrylamide gel electrophoresis
SEM	standard error of the mean
sgRNA	single-guide RNA
siRNA	small interfering RNA
TBST	Tris-buffered saline with Tween 20
TERT	telomerase reverse transcriptase
TSS	transcription start site
