# Supplementary material for: CRISPRi-Mediated Epigenetic Suppression of TERT Reduces Cell Growth in Non-Small-Cell Lung Cancer Cells
Source: Cells. 2026 Jun 24;15(13):1150. doi: 10.3390/cells15131150 (PMC13359582; doi:10.3390/cells15131150)
Supplement: Supplementary file 1 [file cells-15-01150-s001.zip › Cells_TERT_supplementary_revised.pdf]

Supplementary Table S1

qPCR primer	Forward sequence	Reverse sequence
TERT_1	GCCTGAGCTGTACTTTGTC	CGTGTTCTGGGGTTTGATG
TERT_2	TGTCGGAAGCAGAGGTCAG	AGTTGAGCACGCTGAACAGT
GAPDH	GTCTCCTCTGACTTCAACAGCG	ACCACCCTGTTGCTGTAGCCAA

gRNA oligo	Forward sequence	Reverse sequence
TERT_sgRNA_1	caccgGCTGCGCACGTGGGAAGCCC	aaacGGGCTTCCCACGTGCGCAGCc
TERT_sgRNA_2	caccgGGGAGCGCACGGCTCGGCAG	aaacCTGCCGAGCCGTGCGCTCCCc
TERT_sgRNA_3	caccgGCGGTAGTGGCTGCGCAGCA	aaacTGCTGCGCAGCCACTACCGCc
TERT_sgRNA_4	caccgGCTGCGCAGCCACTACCGCG	aaacCGCGGTAGTGGCTGCGCAGCc
TERT_sgRNA_5	caccgCGCCGCACGAACGTGGCCAG	aaacCTGGCCACGTTCGTGCGGCGc
TERT_crRNA_1	aaacGTCCATGTTCACAATCGGCCGCAGCCCG	aaaaCGGGCTGCGGCCGATTGTGAACATGGAC
TERT_crRNA_2	aaacCCCTCTTTTCTCTGCGGAACGTTCTGGC	aaaaGCCAGAACGTTCCGCAGAGAAAAGAGGG
CCR5_sgRNA_1	caccgCCTGCCTCCGCTCTACTCAC	aaacGTGAGTAGAGCGGAGGCAGGc

Supplementary Table S1. Sequences of qPCR primers and gRNA oligos used in this study. Primers and oligos are listed as forward and reverse sequences. gRNA cloning oligos are shown with cloning overhangs in lowercase.

Supplementary Table S2

Gene	Ensembl ID	Base mean	log2 fold change	p-value	FDR (padj)	Significance
ISG15	ENSG00000187608	310.9	8.76×10^{-7}	0.992	1.000	NS
IFIT1	ENSG00000185745	65.5	8.29×10^{-7}	0.807	1.000	NS
DDIT3	ENSG00000175197	221.6	-3.55×10^{-6}	0.316	1.000	NS
ATF3	ENSG00000162772	279.9	-2.58×10^{-6}	0.208	1.000	NS

Supplementary Table S2. Expression of innate immune and cellular stress response genes following sustained TERT repression in H1299 cells (RNA-seq).

Differential expression results for the innate immune markers ISG15 and IFIT1 and the cellular stress markers DDIT3 and ATF3, comparing TERT-targeting versus non-targeting (NT; no-guide dCas9-KRAB) control H1299 cells at day 33 after lentiviral transduction. Values were obtained by DESeq2 analysis; log2 fold changes are shrunken estimates (lfcShrink, apeglm). None of the four genes met the significance criteria (FDR < 0.05 and $|\log2 \text{ fold change}| \geq 1$). NS, not significant.

Supplementary Figure S1

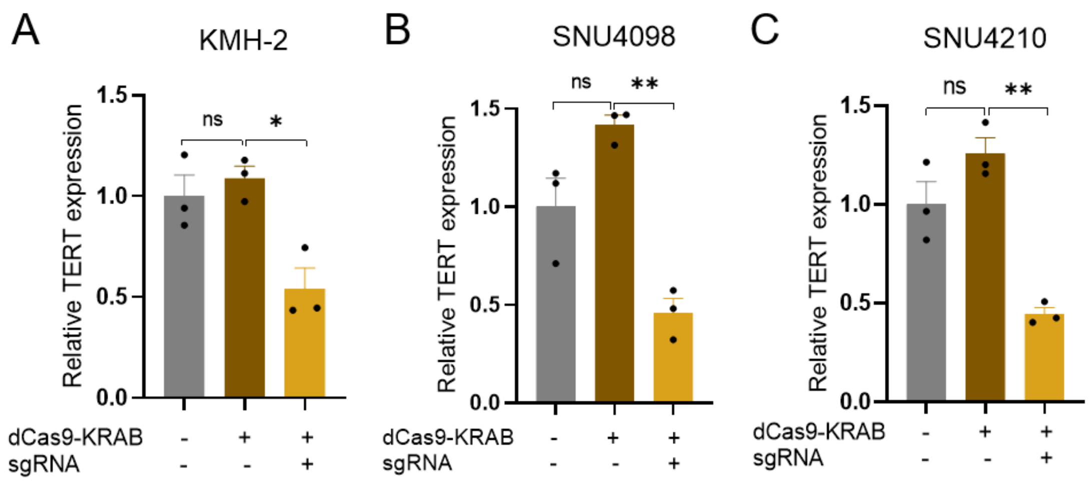

Supplementary Figure S1. RT-qPCR analysis of TERT mRNA levels in KMH-2, SNU-4098, and SNU-4210 cells following dCas9-KRAB mediated repression.

(A–C) TERT mRNA levels in KMH-2 (A), SNU-4098 (B), and SNU-4210 (C) cells were measured by RT-qPCR following dCas9-KRAB mediated repression. KMH-2 is an anaplastic thyroid carcinoma cell line, and SNU-4098 and SNU-4210 are patient-derived glioblastoma cell lines. Expression levels were normalized to GAPDH and presented relative to the mock control, which was set to 1. Data are presented as mean ± SEM from three biological replicates. Statistical significance was assessed by one-way ANOVA followed by a post hoc multiple comparisons test (*$p < 0.05$, **$p < 0.01$).

Supplementary Figure S2

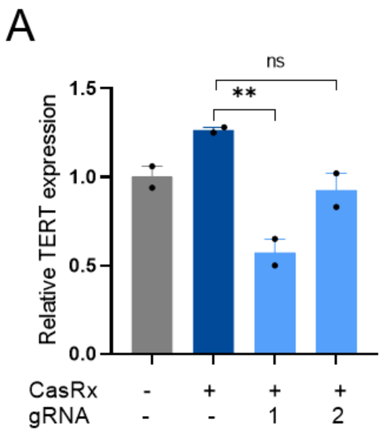

Supplementary Figure S2. Comparison of two CasRx guide RNAs for TERT repression in H1299 cells.

(A) RT-qPCR analysis of TERT mRNA levels in H1299 cells following CasRx-mediated targeting with two independent guide RNAs (gRNA 1 and gRNA 2). TERT mRNA levels were normalized to GAPDH and presented relative to the mock control, which was set to 1. gRNA 1 produced more efficient TERT repression and was selected for subsequent analyses. Data are presented as mean ± SEM from two biological replicates. Statistical significance was assessed by one-way ANOVA followed by a post hoc multiple comparisons test (**$p < 0.01$; ns, not significant).

Supplementary Figure S3

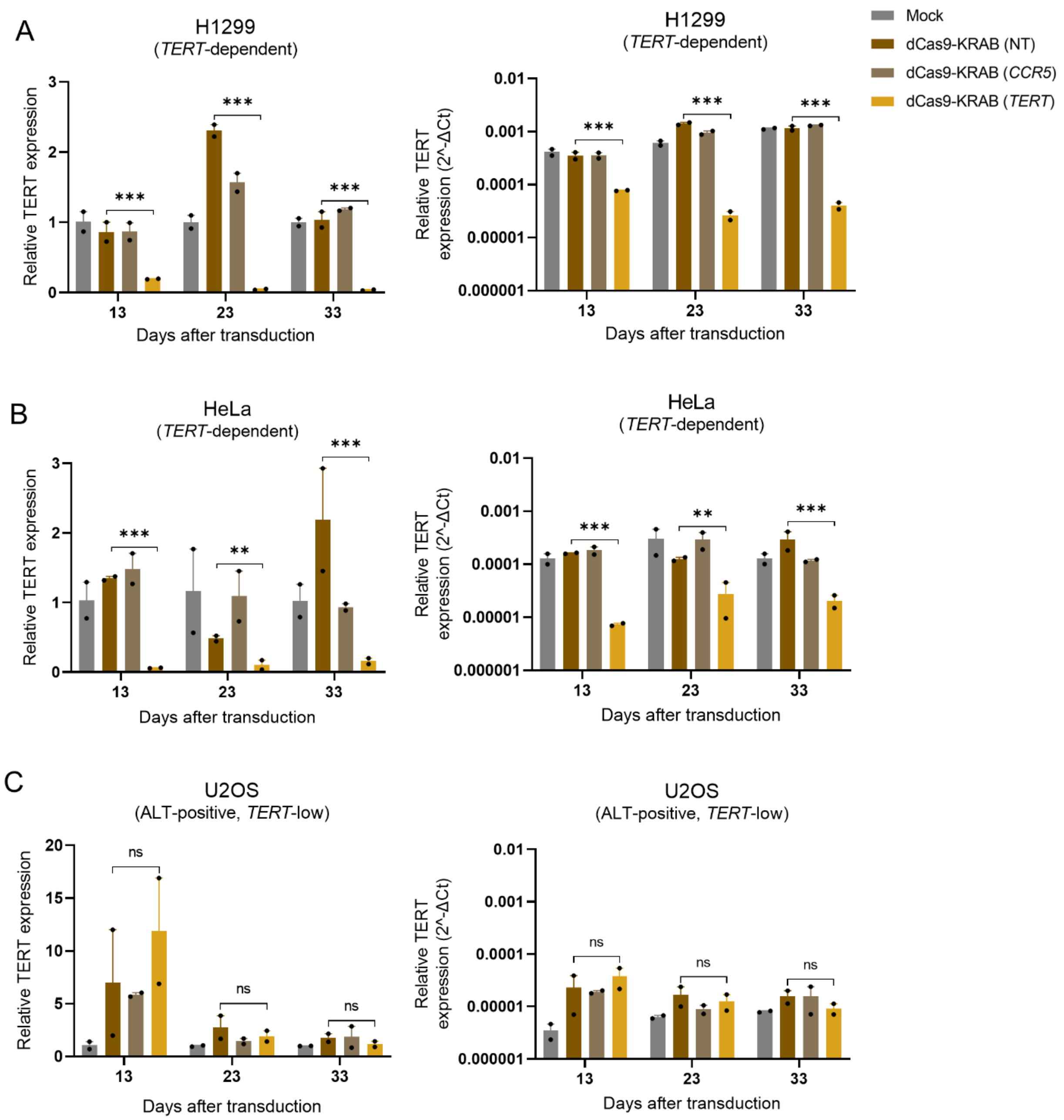

Supplementary Figure S3. Long-term RT-qPCR analysis of *TERT* mRNA expression following lentiviral transduction, presented using two normalization methods.

(A) H1299 cells, (B) HeLa cells, and (C) U2OS cells were analyzed by RT-qPCR at the indicated time points following lentiviral transduction. For each cell line, the left panels show TERT expression normalized to GAPDH and presented relative to the mock group at each time point (set to 1), and the right panels show the same data as $2^{-\Delta Ct}$ values normalized to GAPDH to allow comparison of absolute baseline TERT expression levels across cell lines. NT, non-targeting sgRNA; CCR5, sgRNA targeting CCR5; TERT, sgRNA targeting TERT. Data are presented as mean ± SEM from two biological replicates. Statistical significance was assessed by two-way ANOVA with Dunnett's post hoc multiple comparisons test, comparing the NT control against the TERT-targeting group at each time point (**p

< 0.01, ***p < 0.001; ns, not significant).

Supplementary Figure S4

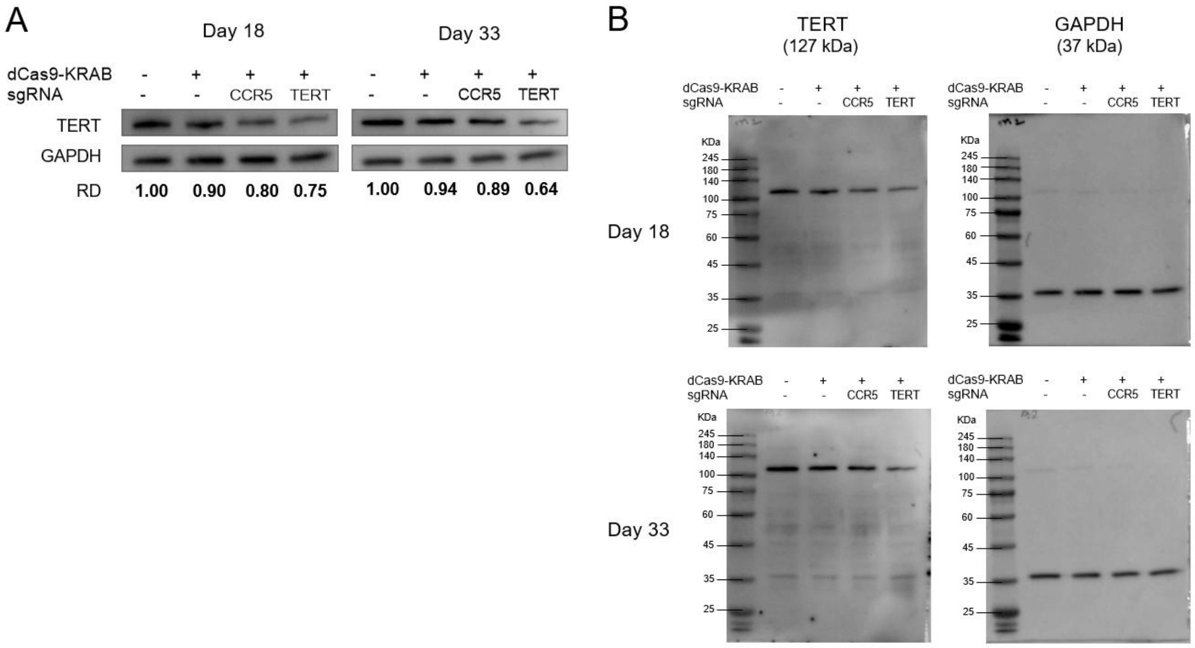

Supplementary Figure S4. Representative immunoblot analysis of TERT protein levels in H1299 cells at Day 18 and Day 33 after lentiviral transduction.

(A) Representative cropped immunoblots of TERT and GAPDH at Day 18 and Day 33. RD (relative densitometric value) is shown below the bands. TERT band intensity was normalized to GAPDH and expressed relative to the mock control, which was set to 1. (B) Uncropped immunoblots corresponding to panel A, showing TERT (127 kDa) and GAPDH (37 kDa) at Day 18 (upper panels) and Day 33 (lower panels).

Supplementary Figure S5

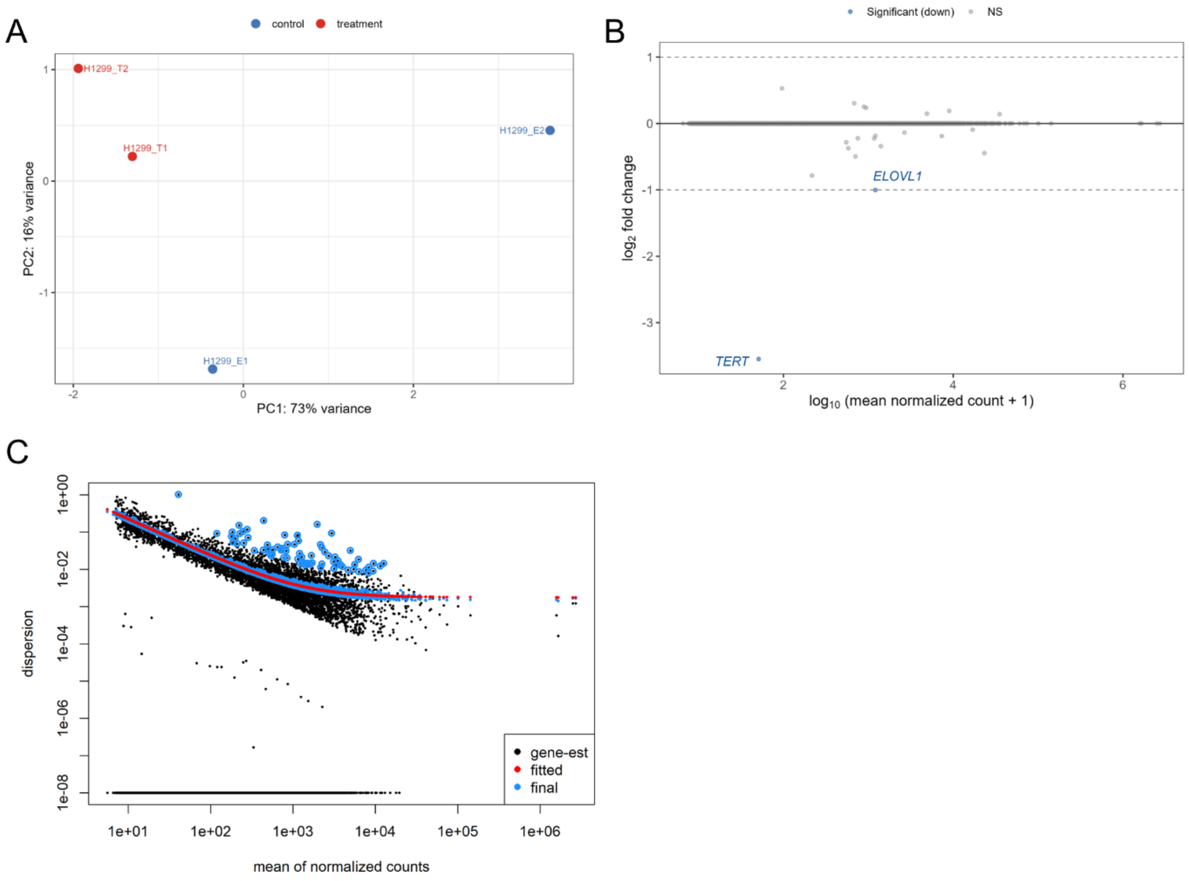

Supplementary Figure S5. RNA-seq quality control of TERT-targeting H1299 cells (NT versus TERT-targeting comparison).

(A) PCA plot comparing NT control and TERT-targeting H1299 cells at day 33 after lentiviral transduction. (B) MA plot of differential gene expression between TERT-targeting and NT control cells. Shrunken log2 fold changes estimated by lfcShrink are shown on the y-axis, and log10 mean normalized counts on the x-axis. Significantly downregulated genes (FDR < 0.05 and |log2 fold change| ≥ 1) are highlighted in blue. (C) Dispersion estimates from DESeq2 for the NT versus TERT-targeting comparison, showing gene-wise estimates (black), fitted values (pink), and final shrunken estimates (blue).

Supplementary Figure S6

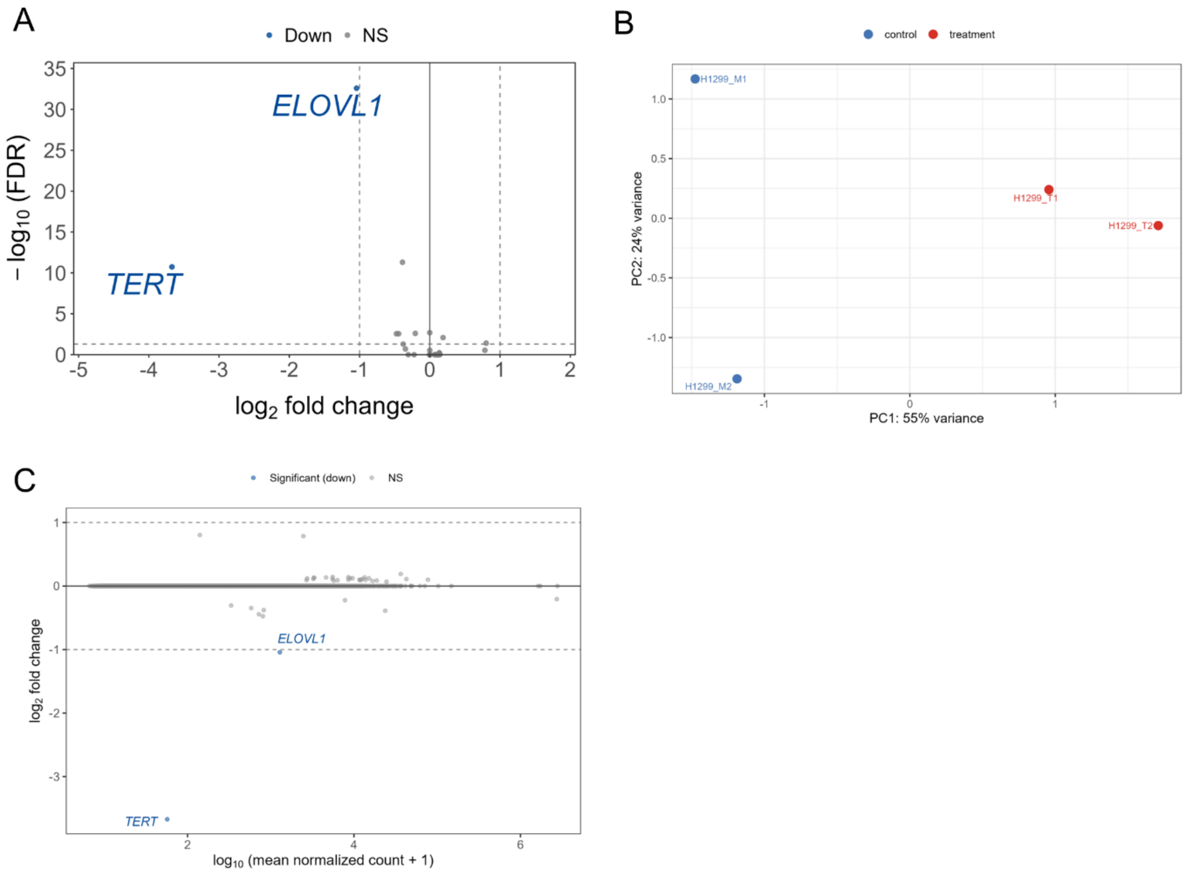

Supplementary Figure S6. Additional transcriptomic analysis of TERT-targeting H1299 cells (Mock versus TERT-targeting comparison).

(A) Volcano plot of differential gene expression between TERT-targeting and Mock cells. Shrunken log2 fold changes estimated by lfcShrink are shown on the x-axis, and −log10(FDR) on the y-axis. Genes with FDR < 0.05 and ∣log2 fold change∣ ≥ 1 were considered significant; blue dots represent significantly downregulated genes, and gray dots represent non-significant genes (NS). (B) PCA plot comparing Mock and TERT-targeting H1299 cells. (C) MA plot of differential gene expression between TERT-targeting and Mock cells, presented as in Supplementary Figure 5B.

Supplementary Figure S7

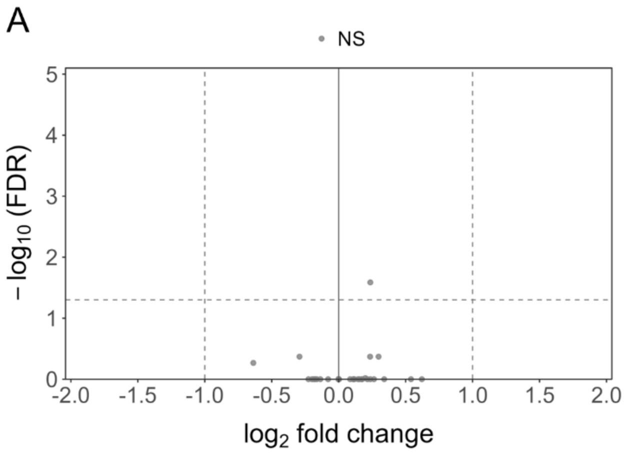

Supplementary Figure S7. Differential gene expression between CCR5-targeting and Mock control H1299 cells.

Volcano plot of differential gene expression between CCR5-targeting sgRNA control and Mock H1299 cells at day 33 after lentiviral transduction. Shrunken log2 fold changes estimated by lfcShrink are shown on the x-axis, and −log10(FDR) on the y-axis. Genes with FDR < 0.05 and |log2 fold change| ≥ 1 were considered significant. No genes met the significance criteria (NS, not significant), indicating that targeting an unrelated genomic locus (CCR5) with the same lentiviral dCas9-KRAB system did not induce detectable transcriptomic changes.

Supplementary Figure S8

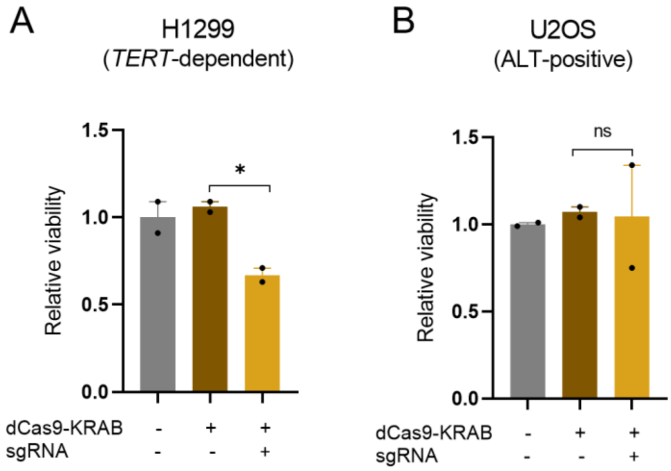

Supplementary Figure S8. CCK-8 viability assay following sustained TERT repression.
Cell viability in H1299 (A) and U2OS (B) cells was measured by CCK-8 assay at Day 18 after lentiviral transduction. H1299 is a TERT-dependent cancer cell line, whereas U2OS is an ALT-positive cell line that maintains telomeres independently of TERT. Data are presented as mean ± SEM from two biological replicates. Statistical significance was assessed by one-way ANOVA followed by Dunnett's post hoc multiple comparisons test (*$p < 0.05$; ns, not significant). Exact adjusted p-values for NT versus TERT-targeting comparisons: H1299, $p = 0.0351$; U2OS, $p = 0.9586$.
